# Air pollution exposure and mammographic breast density in Tehran, Iran: a cross-sectional study

**DOI:** 10.1265/ehpm.22-00027

**Published:** 2022-07-01

**Authors:** Bita Eslami, Sadaf Alipour, Ramesh Omranipour, Kazem Naddafi, Mohammad Mehdi Naghizadeh, Mansour Shamsipour, Arvin Aryan, Mahboubeh Abedi, Leila Bayani, Mohammad Sadegh Hassanvand

**Affiliations:** 1 Breast Diseases Research Center, Cancer Institute, Tehran University of Medical Science, Tehran, Iran; 2 Department of Surgery, Arash Women’s Hospital, Tehran University of Medical Sciences, Tehran, Iran; 3 Department of Surgical Oncology, Cancer Institute, Tehran University of Medical Sciences, Tehran, Iran; 4 Center for Air Pollution Research (CAPR), Institute for Environmental Research (IER), Tehran University of Medical Sciences, Tehran, Iran; 5 Department of Environmental Health Engineering, School of Public Health, Tehran University of Medical Sciences, Tehran, Iran; 6 Noncommunicable Diseases Research Center, Fasa University of Medical Sciences, Fasa, Iran; 7 Department of Research Methodology and Data Analysis, Institute for Environmental Research (IER), Tehran University of Medical Sciences, Tehran, Iran; 8 Department of Radiology, Advanced Diagnostic and Interventional Radiology Research Center, Imam Khomeini Hospital, Tehran University of Medical Sciences, Tehran, Iran; 9 Department of Radiology, Arash Women’s Hospital, Tehran University of Medical Sciences, Tehran, Iran

**Keywords:** Breast density, Mammography, Air pollutants, Nitrogen dioxide, Carbon monoxide

## Abstract

**Background:**

Air pollution is one of the major public health challenges in many parts of the world possibly has an association with breast cancer. However, the mechanism is still unclear. This study aimed to find an association between exposure to six criteria ambient air pollutants (PM_2.5_, PM_10_, SO_2_, NO_2_, O_3_, and CO) and mammographic breast density (MBD), as one of the strongest predictors for developing breast cancer, in women living in Tehran, Iran.

**Methods:**

Participants were selected from women attending two university hospitals for screening mammography from 2019 to 2021. Breast density was rated by two expert radiologists. Individual exposures to 3-year ambient air pollution levels at the residence were estimated.

**Results:**

The final analysis in 791 eligible women showed that low and high breast density was detected in 34.8 and 62.2 of participants, respectively. Logistic regression analysis after considering all possible confounding factors represented that an increase in each unit of NO_2_ (ppb) exposure was associated with an increased risk of breast density with an OR equal to 1.04 (95CI: 1.01 to 1.07). Furthermore, CO level was associated with a decreasing breast density (OR = 0.40, 95CI = 0.19 to 0.86). None of the other pollutants were associated with breast density.

**Conclusion:**

Higher MBD was associated with an increased level of NO_2_, as a marker of traffic-related air pollution. Furthermore, CO concentration was associated with a lower MBD, while other criteria air pollutants were not related to MBD. Further studies are needed to evaluate the association between ambient air pollutants with MBD.

**Supplementary information:**

The online version contains supplementary material available at https://doi.org/10.1265/ehpm.22-00027.

## Background

Air pollution is one of the major public health challenges in many parts of the world and in large cities. Ambient air pollution is a mixture of different pollutants originating from natural and anthropogenic sources and the International Agency for Research on Cancer (IARC) classified it as Group 1 carcinogenic to humans [1]. Several studies showed that short- and long-term exposure to air pollution can cause many chronic and acute health effects. Numerous studies reported that long-term exposure to outdoor air pollution caused globally around 4.24 million premature deaths annually [2–5].

Breast cancer is one of the worldwide leading causes of mortality and morbidity, and according to a report of GLOBOCAN in 2018 accounts for more than 11.6 of all female cancers; while the disease burden of breast cancer shows an increasing trend in some populations [6]. Because this disease imposes a heavy burden on the health system, more preventive efforts are necessary and further investigation should explore the underlying reasons for these epidemiological trends.

Ecologic studies suggest that breast cancer risk is elevated in urban areas with high levels of air pollution compared to rural areas [7, 8]. Air pollution contains many carcinogens and other compounds that may act as endocrine disruptors, and air pollution exposure has been globally linked to many cancers such as lung, breast, and bladder cancer [9]. In 1979 Hill and Winder found that inhaled toxicants (nicotine and cotinine) were detectable in breast fluid after 30 minutes of smoking [10]. Thus, toxic chemicals can reach the breast tissue and have possibly some impacts on it.

A systematic review and meta-analysis in 2021 investigated whether high levels of air pollution exposure were related to increased breast cancer risk [11]. This study showed that NO_2_ had a “moderate level of evidence” and that PM_2.5_ and PM_10_ have an “inadequate level of evidence” for supporting their association with breast cancer risk. Also, the biological mechanism of the effects of air pollutants on breast cancer still remains unknown [11].

Mammographic breast density (MBD) is one of the strongest predictors and biomarkers for breast cancer [12]. Limited studies evaluated the association between MBD and air pollution exposure, which had also inconsistent results [13–16]. To draw risk-reducing strategies for breast cancer, studying the impacts of ambient air pollutants on breast density may provide valuable data. Further studies have been recommended, due to study limitations in the exposure assessment, adjusting confounding variables, and outcome ascertainment [11].

Tehran the capital of Iran is a megacity with about 10 million residents and air pollution is a major environmental challenge in this city. Tehran with an altitude of 1000–1800 meters above the mean sea level is located in a valley and is surrounded on the north, northwest, east, and southeast by medium-high to high Alborz mountain ranges. The climate is semi-arid with a lack of wind and low annual precipitation. Tehran’s geographical and climate situation causes trapping air pollution within the city, especially during winter. The people of Tehran are exposed to high levels of ambient air pollution, to the point where government and non-government offices are sometimes closed due to the severity of air pollution [3, 17]. Therefore, the present study was designed to investigate whether there is an association between exposure to six criteria ambient air pollutants (Nitrogen dioxide (NO_2_), Sulfur dioxide (SO_2_), Carbon monoxide (CO), Ozone (O_3_), and Particulate matter (PM) _2.5_, _10_) and MBD in women living in Tehran, Iran.

## Methods

### Study design and participants

This study was designed as a cross-sectional study; participants were selected from women attending two university hospitals affiliated to Tehran University of Medical Sciences, Tehran, Iran, for screening mammography from 2019 to 2021. The study was approved by the ethics committee of Tehran University of Medical Sciences (IR.TUMS.VCR.REC.1398.897), and all participants have signed informed consent. All methods have been performed in accordance with the relevant principles of the Declaration of Helsinki.

Criteria for inclusion in the study were at least 3 years of residency in the capital city of Iran (Tehran) and having the ability to fill questionnaires. Exclusion criteria included suspicion for malignancy in the current mammography and an imprecise address.

### Data collection

Participants were asked to fill out a questionnaire that captured demographic information, self-reported age, weight, height, reproductive history, menopause status, smoking history (active and passive), history of oral contraceptive (OCP) use, current use of hormone replacement therapy, and familial history of breast and ovarian cancer. All women who either had a current or previous history of active and passive (secondhand) smoking were defined as having a positive exposure to smoke. Menopause was defined as cessation of the menstrual period at least one year sooner; women were stratified into premenopausal and postmenopausal status. Furthermore, we gathered information about current aspirin and metformin use and consumption duration in each woman. Routine use of supplements including vitamin D, calcium, Vitamin E, Omega 3, and Evening Primrose oil were also recorded.

One expert radiologist reported the breast density in each center. In order to evaluate the agreement between the radiologists’ reports, the third independent radiologist was rated the mammographic breast density of the same cases. Radiologists rated MBD according to the American College of Radiology (ACR) Breast Imaging-Reporting and Data System (BI-RADS) classification into four categories: almost entirely fatty (BI-RADS a), scattered areas of fibro glandular density (BI-RADS b), heterogeneously dense (BI-RADS c), and extremely dense (BI-RADS d) [18]. We categorized MBD into low density (a and b) and high density (c and d).

The exact address of residence of the participants in the recent 3 years and the telephone number of that place were recorded. Also, in the employed women, the address of their place of work and the hours of their attendance to work were recorded.

For sample size calculation, since the association between MBD and air ambient pollutants, has been investigated in limited studies which had inconsistent results, we expected 40% exposure to high ambient air pollutants in low breast density with assumed odds ratio (OR) equal to 1.5. Therefore, we calculated that about 800 samples would be required to find any possible association between pollutants and MBD with a power of 80% and α = 0.05 by using the Epi Info website (www.cdc.gov/epiinfo).

### Air pollution exposure assessment

In this study, estimating the exposure of participants to ambient criteria air pollution was done in the following three steps:

1. Outdoor air quality data gathering from fixed monitoring stations belong to Tehran Air Quality Control Company.
2. Data cleaning of air quality monitoring stations in order to outlier data detection.
3. Individual long-term exposure assessment using air quality data and inverse distance weighting (IDW) approach.

### Air quality data gathering

Real-time hourly ambient air quality in Tehran city is monitored by fixed monitoring stations. In Tehran city, Air Quality Control Company (AQCC) affiliated with the Tehran Municipality is responsible for monitoring criteria air pollutants (PM_2.5_, PM_10_, NO_2_, O_3_, SO_2_, and CO). At the time of this study in 2020, there were 22 monitoring stations in Tehran that belonged to the AQCC. Considering that the data of the AQCC stations are available on an hourly basis and are publicly available online and that these monitoring stations are spatially located in all districts of Tehran city, therefore we used the data obtained from air quality monitoring stations belonged to AQCC in this study. Air quality monitoring stations in Tehran city are measured ambient PM_2.5_ and PM_10_, NO_2_, O_3_, CO, and SO_2_ by using the beta-attenuation (Met One BAM-1020, USA; and Environment SA, MP 101 M, France), chemiluminescence (Ecotech Serinus 40 Oxides of Nitrogen Analyzer, Australia), UV-spectrophotometry (Ecotech Serinus 10 Ozone Analyzer, Australia), non-dispersive infrared absorption (Ecotech Serinus 30 carbon monoxide Analyzer, Australia), and ultraviolet fluorescence (Ecotech Serinus 50 SO_2_ Analyzer, Australia) methods, respectively [19].

Finally, hourly data of six outdoor criteria air pollutants for the 3-year residency of participants were obtained from the website of AQCC (Available at: http://airnow.tehran.ir/home/DataArchive.aspx).

### Air quality data processing

Data quality control is the most important part of air quality studies and estimating health effects. Data quality assurance was performed according to international organization guidelines such as World Health Organization (WHO), Environmental Protection Agency (EPA), and the European Union [20–22]. Due to numerous operational and calibration problems related to air pollutant measuring stations, outlier detection and data cleaning from monitoring stations is very important and the results would have insufficient scientific validity if this step is omitted.

In the present study, first, the data of all monitoring stations were obtained and then hourly data coverage of each pollutant in each station during the three years was determined. Included monitoring stations were only stations with ≥75 completeness of the total hours during the study period [20, 22]. Then, in order to outlier data detection, the modified Z-score approach proposed by some researchers for this purpose was used [3, 21, 23, 24]. Briefly, in order to identify outlier data, the following steps were used:

Calculating the Z score for each hourly data at each station using the following equation:

$$Z=\frac{(\text{Concentration}{)}_{\text{Hourly}}-(\text{Concentration}{)}_{\text{Annual}}}{\text{SD of annual concentrations}}$$

Calculating the following four conditions:

$$\begin{array}{rl} Z_{1} & =|Z|>4\\ Z_{2} & =(Z_{t}-Z_{t-1})>6\\ Z_{3} & =\left(\frac{Z_{t}}{RM3(Z_{t})}\right)>1.5\\ Z_{4} & =\frac{\left(Z_{t}-Z_{t-1}\right)}{\text{City}(Z_{t}-Z_{t-1})}>2 \end{array}$$

Finally, the air quality data were detected as outlier data and removed if they meet the four above-mentioned conditions. By using these criteria, 5 air quality monitoring stations were excluded from exposure assessment. On the other hand, air quality monitoring stations with ≥75 reliable hourly data coverage during the study period were 17 ones.

### Individual long-term exposure assessment

To determine the long-term exposure of each participant to ambient air pollutants, the exact address according to the area, place, street, and alley in each year was obtained. In working women, if the place of work and living were different, the area and time spent in that area were also considered. Then, according to the location of monitoring stations and the location of the study subject, three of the nearest included air quality monitoring stations were identified for each participant, and using the average annual data and IDW method, the 3-year annual mean of exposure was estimated as long-term exposure for each study subject.

### Statistical analysis

Cohen’s kappa (κ) was run to determine if there was an agreement between two radiologists on breast density in the reports of the same case. Data were presented with mean ± standard deviation (SD) for continuous and frequency (percentage) for categorical variables. The ANOVA, t-test, and chi-square test were used to compare variables between study groups in the univariable analysis step. A multiple logistic regression analysis was done between all pollutants criteria as independent variables with breast density as a dichotomous dependent variable (low = 0 and high = 1). Because the pollutants criteria had a high inter-correlation with each other and the multivariable regression could be affected by the collinearity problem, the number of independent variables was reduced in order to solve this problem. So, we ran two multivariable logistic regressions, non-stepwise and stepwise algorithms and the result of the stepwise algorithm was chosen as the final results. After that, another logistic regression analysis was done to evaluate whether the effects of pollutants on breast density were independent or affected by potential confounding variables. This analysis was done as three models. For the first one, only significant pollutants were considered in the model. In the second model, in addition to pollutants, medical (history of breast disease, menopause statues, history of OCP use, and parity) and demographic (age, BMI, and smoking) variables were considered. In the last model, the history of medicine and supplement use (metformin, aspirin, vitamin D, calcium) was added to the previous variables. In the logistic regression analysis, an OR with a 95% confidence interval (CI) was reported in addition to the p-value. All calculations were performed in IBM SPSS (IBM Corp. Released 2019. IBM SPSS Statistics for Windows, Version 26.0, Armonk, NY: IBM Corp) and the charts were drawn with MS Excel (Microsoft Co., Redmond, WA, USA). P-value < 0.05 was considered significant.

## Results

The response rate of women to participate in this study was 98%. Based on inclusion criteria 813 women were screened in this study. We excluded participants who were suspicious for malignancy in the current mammography (n = 14), and who had written an incomplete address (n = 8); finally, 791 eligible women were recruited. The mean age was 50.14 ± 7.61 (38–80) years old. About half of the women (50.1) were premenopausal, and half of them were in menopause (49.9) at time of the recruitment in the study.

There was almost perfect agreement between the radiologists’ report, κ = 0.979 (95CI: 0.965 to 0.993; p < 0.001). In the mammographies, low breast density was reported in 34.8 (n = 299) and high breast density in 62.2 (n = 492). Table 1 compares general and reproductive factors and other variables between breast density categories. As shown, all variables except the age of menarche and the whole breastfeeding duration had a statistically significant difference between the two groups of breast density (P-value < 0.05). The comparison between the 4 categories of MBD is presented in Supplementary Table 1.

**Table 1:** Demographic, medical and drug history of women with high and low mammographic breast density.

**Variables**		**Low density** **(n = 299)**	**High density** **(n = 492)**	**P-value**
**Age** (years)		53.25 ± 8.29	48.25 ± 6.47	**<0.001**

**Body mass index** (Kg/m^2^)		29.80 ± 5.35	27.19 ± 4.16	**<0.001**

**Age of menarche** (years)		13.69 ± 1.57	13.51 ± 1.49	0.359

**Age at first birth** (years)		21.32 ± 5.22	22.46 ± 5.32	**0.006**

**Parity** (n)		2.59 ± 1.58	1.96 ± 1.30	**<0.001**

**Breastfeeding duration** (months)		35.14 ± 32.48	32.76 ± 29.30	0.290

**Menopause**	No	93 (23.5)	303 (76.5)	**<0.001**
	Yes	206 (52.2)	189 (47.8)	

**History of OCP**	No	160 (32.6)	331 (67.4)	**<0.001**
	Yes	139 (46.3)	161 (53.7)	

**Smoking**	No	261 (36.5)	455 (63.5)	**0.016**
	Active or passive	38 (50.7)	37 (49.3)	

**Occupation**	Housewife	264 (39.3)	407 (60.7)	**0.008**
	Employed	20 (23)	67 (77)	
	Retired	15 (45.5)	18 (54.5)	

**Metformin**	No	250 (35.7)	450 (64.3)	**0.001**
	Yes	49 (53.8)	42 (46.2)	

**Aspirin**	No	239 (34.9)	446 (65.1)	**<0.001**
	Yes	60 (56.6)	46 (43.4)	

**Calcium**	No	143 (32.5)	297 (67.5)	**0.001**
	Yes	156 (44.4)	195 (55.6)	

**Vitamin D**	No	164 (41.2)	234 (58.8)	**0.047**
	Yes	135 (34.4)	258 (65.6)	

**Vitamin E**	No	247 (38.3)	398 (61.7)	0.547
	Yes	52 (35.6)	94 (64.4)	

**Evening Primrose oil**	No	287 (38.2)	465 (61.8)	0.353
	Yes	12 (30.8)	27 (69.2)	

**Omega-3**	No	264 (37.9)	433 (62.1)	0.904
	Yes	35 (37.2)	59 (62.8)	

**History of Breast Disease**	No	217 (35.6)	392 (64.4)	**0.021**
	Yes	82 (45.1)	100 (54.9)	

Continuous variables present as mean ± standard deviation and categorical variables present as number with percentages in parenthesis. P-values were computes with t test for continues and chi square test for categorical variables.

**Fig. 1:**
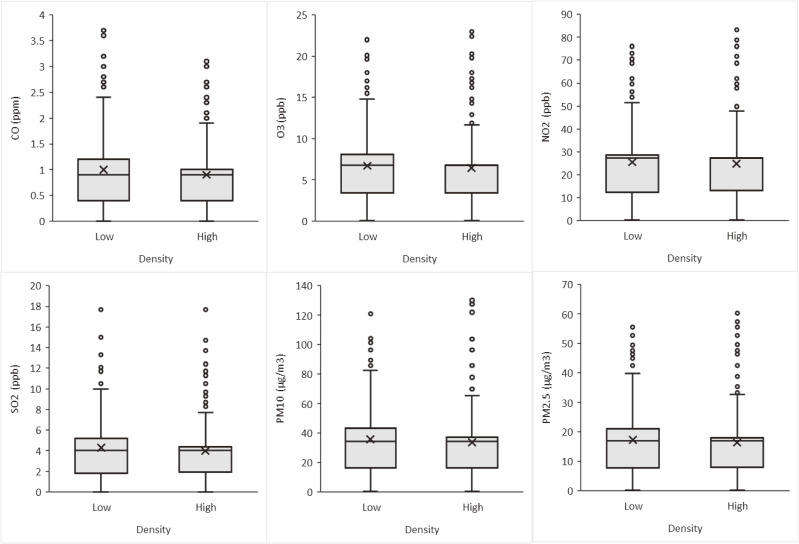
Comparison of participants’ exposure to ambient air pollutants with high and low mammographic breast density. The P-values of the t-tests were: P-value = 0.054 (CO), P-value = 0.404 (O_3_), P-value = 0.601 (NO_2_), P-value = 0.125 (SO_2_), P-value = 0.233 (PM_10_), and P-value = 0.295 (PM_2.5_). Further information is presented in Supplementary Table 2.

In the first step, in a univariate analysis using a t-test, all six pollutants criteria were compared between low and high breast density. In this comparison, except for ambient air CO, which was on the borderline statistically significant (P-value = 0.054), other variables were not significantly different between the two groups (Fig. 1 & Supplementary Table 2). Due to an unclear trend of ambient criteria air pollutants between the four categories of MBD, the comparison has been conducted only between high and low breast densities; and the comparison between the 4 categories is presented in Supplementary Table 3.

Unlike univariate analysis, multiple regression analysis between six ambient air pollutants and MBD showed that outdoor air NO_2_ (P-value = 0.003) and CO (P-value = 0.001) had a significant relationship with breast density. Logistic regression analysis with stepwise algorithm and breast density as a dependent variable showed that an increase in each unit of NO_2_ (ppb) exposure was associated with an increased risk of breast density with an OR equal to 1.04 (95 CI: 1.01 to 1.07); and an OR equal to 1.47 (95CI: 1.10 to 1.97) for each 10 unit increase in NO_2_. Furthermore, CO level was associated with a decreasing risk of breast density in each 1 ppm (OR = 0.33, 95 CI = 0.17 to 0.64). None of the other pollutants were associated with breast density (Table 2).

**Table 2:** Evaluation the impact of pollutants on mammographic breast density with stepwise and non-stepwise logistic regression.

	**Non-stepwise algorithm**				**Stepwise algorithm**			

	**P-value**	**OR**	**95C.I Lower**	**95C.I Upper**	**P-value**	**OR**	**95C.I Lower**	**95C.I Upper**
**CO** (ppm)	**0.024**	0.429	0.206	0.893	**0.001**	0.331	0.172	0.637
**NO_2_** (ppb)	**0.055**	1.045	0.999	1.093	**0.003**	1.039	1.013	1.066
**O_3_** (ppb)	0.870	0.988	0.852	1.145				
**SO_2_** (ppb)	0.605	0.926	0.692	1.240				
**PM_10_** (µg/m^3^)	0.378	0.944	0.829	1.073				
**PM_2.5_** (µg/m^3^)	0.305	1.129	0.896	1.422				
**Constant**	<0.001	1.754		—	<0.001	1.758	—	

OR = Odds ratio, C.I = Confidence interval, NO_2_ = Nitrogen dioxide (NO_2_), SO_2_ = Sulfur dioxide, CO = Carbon monoxide, O_3_ = Ozone, PM = Particulate matter.

In order to evaluate whether the effects of pollutants on MBD are independent or disappear under the influences of confounding variables, two other multiple analyses were performed. In the first model, basic and reproductive factors (age, body mass index (BMI), Smoking, history of OCP usage, parity, menopause, and history of breast disease) were entered into the model. In the second model, metformin and aspirin intake, vitamin D, and calcium consumption were also entered into the model. Table 3 illustrates the results of the three models. Finally, multiple logistic regression analysis showed that ambient air CO (P = 0.018) and NO_2_ (P = 0.022) had independent effects on breast density.

**Table 3:** Logistic regression models for ambient air pollutants impact on mammographic breast density considering confounder variables.

		**P-value**	**OR**	**95 C.I. for OR**	

				**Lower**	**Upper**
**Model 1**	CO	**0.001**	0.331	0.172	0.637
	NO_2_	**0.003**	1.039	1.013	1.066
	Constant	<0.001	1.758		

**Model 2**	CO	**0.020**	0.411	0.195	0.868
	NO_2_	**0.026**	1.034	1.004	1.064
	Age	0.000	0.942	0.915	0.971
	BMI	0.000	0.884	0.851	0.919
	Smoking	0.063	0.602	0.353	1.029
	History of OCP	0.000	0.526	0.375	0.738
	Menopause	0.002	0.522	0.348	0.782
	Parity	0.708	0.975	0.856	1.111
	History of Breast disease	0.106	0.728	0.495	1.070
	Constant	<0.001	2454.3		

**Model 3**	CO	**0.018**	0.404	0.190	0.856
	NO_2_	**0.022**	1.035	1.005	1.066
	Age	0.000	0.947	0.919	0.976
	BMI	0.000	0.884	0.851	0.920
	Smoking	0.088	0.624	0.363	1.072
	History of OCP	0.000	0.536	0.381	0.753
	Menopause	0.004	0.544	0.360	0.823
	Parity	0.938	0.995	0.872	1.135
	History of Breast disease	0.124	0.735	0.496	1.088
	Metformin	0.726	0.913	0.547	1.524
	Aspirin	0.070	0.641	0.397	1.037
	Vitamin D	0.040	1.426	1.016	2.001
	Calcium	0.203	0.795	0.558	1.132
	Constant	<0.001	1710.8		

Model 1, only significant pollutants, Model 2, significant pollutants with medical (history of breast disease, menopause statues, history of OCP use, and parity) and demographic (age, BMI, and smoking) risk factors, Model 3, significant pollutants with medical and demographic risk factors also with medicine and supplement use (metformin, aspirin, vitamin D, calcium). All models were non stepwise multivariable logistic regression. OR = Odds ratio, C.I = Confidence interval, NO_2_ = Nitrogen dioxide (NO_2_), SO_2_ = Sulfur dioxide, CO = Carbon monoxide, O_3_ = Ozone, PM = Particulate matter.

In addition, a separate analysis was performed considering menopausal status. The relationship between ambient air NO_2_ (OR = 1.04, 95 CI: 1.002–1.077, P-value = 0.039) and CO (OR = 0.31. 95 CI: 0.125–0.785, P-value = 0.013) with MBD was observed only in menopausal women in the same direction as stated. In premenopausal women, breast density was not associated with ambient air pollutants (Supplementary Table 4). The last analysis brought up menopause status as a moderator of the relationship between exposure to air pollution and breast density. A similar analysis, taking into account age, found that age as a moderator did not significantly change the relationship between pollutants and breast density (Supplementary Table 5).

The comparison in breast density in women who live a lifetime in Tehran (n = 420, 53.1) and other women didn’t show any significant difference (data not shown in table).

## Discussion

The present study has evaluated the precise impact of long-term exposure to six criteria ambient air pollutants on MBD in Iranian women for the first time. Actually, many known and unknown factors are involved in breast tissue changes and eventually in breast cancer and it’s not possible to control all confounding factors in a single context. By the way, based on the available evidence, we tried to evaluate the effects of six criteria ambient air pollutants on breast density considering the factors that seem to have an impact on MBD (basic and reproductive factors, aspirin, metformin, and supplement intake). To the best of our knowledge, there is no study with this broad level of assessment.

Our results represented that outdoor air NO_2_ and CO exposure had statistically significant impacts on MBD. We found that an increased level of NO_2_, as a marker of traffic-related air pollution [25], is associated with a higher MBD. Furthermore, ambient air CO concentration was associated with a lower MBD, while other criteria air pollutants were not related to MBD. Our present results about ambient air NO_2_ and PMx (PM_2.5_ & PM_10_) concentration were consistent with a recent systematic study and meta-analysis that found an increased risk of breast cancer with an increase in each 10 unit in NO_2_ exposure (Hazard ratio (HR) = 1.02, 95 CI = 1.01–1.04), while PM_2.5_ and PM_10_ revealed no statistically significant associations with breast cancer risk [11]. The results of our study on the relationship between air pollutants and MBD seem to be in line with studies that have examined the relationship between these pollutants and breast cancer.

Limited studies have evaluated the association between criteria ambient air pollutants and MBD with inconsistent results [13, 14, 16]. Similar to our study, Du Pre and their colleague’s results in the Nurses’ Health Study didn’t support that recent exposure to particulate matter (PM_2.5_, PM air_2.5-10_, PM_10_) influenced breast density [13]. Two other studies had contradictory results with the present study [14, 16]. The Danish Diet, Cancer and Health Cohort investigated the association between long-term exposure to traffic-related air pollution (NO_2_, NO_x_) and MBD in a prospective cohort of women aged 50 and older. They found a reverse association between air NO_2_ level and MBD (OR = 0.89, 95 CI: 0.80–0.89 per 10 µg/m^3^) with no interaction with menopause, smoking, or obesity [14]. In the Yaghjyan et al. study, women older than 40 years old with known residential zip codes and estimated PM_2.5_ and O_3_ levels for the year preceding the mammogram date were included. They found that women with extreme breast density had higher mean PM_2.5_ and lower O_3_ exposure levels [16].

Numerous studies in line with our study have investigated the relationship between endocrine-disrupting chemicals (EDCs) and heavy metals with MBD [15, 26, 27]. In a cross-sectional study in 725 women (40–65 years old), a higher urinary level of magnesium was associated with a higher MBD [26]. In postmenopausal women (n = 264), women with high serum levels of BPA and mono-ethyl phthalate had an elevated breast density [27]. In a large-scale study (n = 222,581), the relation of the MBD of women who underwent a routine screening mammogram in 2011 and residential levels of ambient air polycyclic aromatic hydrocarbons (PAHs) and metals was assessed. Higher residential levels of arsenic, cobalt, lead, manganese, nickel, or PAHs were individually associated with breast density. Comparing the highest to the lowest quartile, higher odds for dense breasts were observed for cobalt (OR = 1.60, 95 CI 1.56–1.64) and lead (OR = 1.56, 95 CI 1.52–1.64). These associations were stronger in premenopausal women [15]. An exception is one cross-sectional study of PCBs, which reported some PCB congeners’ plasma levels were associated with lower MBD in postmenopausal women [28].

In the present study, the relationship between ambient air NO_2_ and CO with MBD was observed only in menopausal women in the same direction as stated and in premenopausal women, breast density was not associated with ambient air pollutants. These findings should confirm in a large population study, because the impact of environmental pollutants on breast cancer may be greater during several windows of susceptibility (WOS) in women’s life such as pregnancy, puberty, and the menopausal transition [29]. Therefore, further studies which focus on pollutants exposure in these specific periods cause the understanding of the etiology of breast cancer. In this study, we evaluate the effect of menopause and age as moderator variables which could change the size or direction of the relationship between other variables. Our results showed that breast density correlated with air pollution only in menopausal women, which means that the relationship between exposure to air pollution and breast density was changed by menopause status. So, after rejecting the same effect for age, we can claim that menopause status is a moderator variable.

Consistent with the present study, a review study by White and colleagues that summarized eight case-control studies and nine cohort studies suggested little evidence to support an association between particulate matter and breast cancer risk. More consistent findings have reported a relation between NO_2_ or NO_X_ level and breast cancer [30].

This study has provided an interesting finding on the reverse association between MBD and CO. Since we couldn’t find any studies that examine the association between MBD and CO and there is a positive correlation between MBD and breast cancer, we have mentioned studies that evaluated the effects of CO on breast cancer. Two recent studies evaluated the effect of CO on breast cancer had equivocal results. A Korean study reported that CO concentration was positively and significantly associated with breast cancer (OR = 1.08, 95 CI = 1.06–1.10) [31] and another cohort study in Taiwan found that women who had CO poisoning (COP) were at a lower risk of developing breast cancer than those without COP [32]. Growing evidence has revealed the toxic effect of CO causes cancer cells death due to severe hypoxia that may play a role in tumor progression before the resistance of tumor cells to a hypoxic environment is developed [33]. Furthermore, it inhibits the proliferation of human cancer cells and increases the mice’s survival rate [34]. CO application for cancer treatment is an emerging hope, and a number of novel CORMs, a group of transition metal carbonyls or boranocarbonates that can release CO upon transformation, are recently used as an anticancer treatment for different cancers as well as breast cancer [35]. In this regard, the results of our study on the inverse relationship between CO and breast density can lead to the hypothesis that CO exposure may reduce the risk of breast cancer through the mechanism inhibiting increasing breast density. This hypothesis should be tested in vivo and in vitro studies.

Our results show that some of the risk factors of breast cancer such as age, BMI, and smoking had the reverse association with breast density. These findings are debatable because the evidence showed breast density is a strong risk factor for breast cancer, independent of age and other risk factors, is highly heritable, and has the properties of a quantitative trait [36]. Therefore, in an analysis of the association between MBD and the risk of breast cancer, adjustment for age, BMI, as well as other well-known risk factors is recommended. Furthermore, the known risk factors for breast cancer explain only 20–30% of the variance in mammographic density [37]; most of them are explained by genetic factors. However, the exact mechanism of the effect of MBD on breast cancer still remains unknown.

Because the probability of developing breast cancer increases with age, declining the prevalence of MBD that occurs with increasing age could seem a paradox. However, Pike and Colleagues’ model explained the rate of breast-tissue aging, rather than chronological age, is the relevant measure for describing the age-specific incidence of breast cancer [38]. Finally, it should be noted that MBD reflects the cumulative exposure to other factors such as hormonal and growth factors that stimulate cell division in breast stroma and epithelium, which could be other important factors underlying the age-specific incidence of breast cancer [36].

The advantage of this study is, we considered all the possibly effective factors and known determinants of MBD, all of which are estrogen-related. As it was demonstrated in Table 1, 91 (11.5) and 106 (13.4) women in the present study sample consumed metformin and aspirin, respectively. Numerous studies have evaluated the effects of aspirin and metformin on MBD with inconsistent results [39–42]. In addition, in the present study, we found a marginally significant increasing breast density with vitamin D intake (p-value = 0.047). The association between vitamin D and MBD remains poorly understood in many studies [43–45] due to differences in study designs, MBD assessment, vitamin D exposure assessment methods, categorization of women with some variables such as menopause, lack of attention to seasonal variation, diet, and other considerations. Since the vitamin D intake in this study was based on the self-reported of women and finding an association between vitamin D intake and breast density was not our goal, this result may be crude and inaccurate. Anyway, this factor has been considered by researchers as an influential factor (in a positive or negative direction) on breast density.

According to the findings of the mentioned studies and the high percentage of women who had taken metformin (11.5), aspirin (13.4), vitamin D (49.7), and calcium (44.4) in our study, it seems that without considering the use of these drugs, the results may not be expressed correctly. However, our findings showed that even by considering these factors, the results did not change.

Our study had some limitations. The primary limitation is due to sampling type that the temporal link between the outcome and the exposure cannot be determined. Another point is that despite the high sample size, the generalizability of the study may be questionable. Due to the ultrasound device in our center was not able to assess the percent density of the breast, we couldn’t report the exact percentage of breast density as well as the cut-off point level. It is important to note that most of the sampling in this study coincided with the worldwide onset of the COVID-19 pandemic. Therefore, it is possible that the participants during this period were women at higher risk of breast cancer, who had been referred for screening despite the COVID-19 pandemic. In this study, since our sampling area was two public hospitals and women were homogenous from the view of socioeconomic status and the majority of them belong to low to middle SES, we didn’t collect information on socioeconomic status. Therefore, we assume that this factor was not a significant confounder in our study.

Considering our results and other evidence, in highly air polluted areas, perhaps MBD monitoring as an available tool in each population, can help the prediction of future breast cancer occurrence. Further studies are necessary to find the prevalence of breast cancer in highly polluted geographic areas.

## Conclusion

In conclusion, higher MBD was associated with an increased level of NO_2_, as a marker of traffic-related air pollution. Moreover, air CO concentration was associated with a lower MBD, while other criteria air pollutants were not related to MBD. Further studies are needed to evaluate the association between ambient air pollutants especially CO level as well as other pollutants with MBD.

## Abbreviations

MBD: Mammographic breast density
IARC: International Agency for Research on Cancer
BPA: Bisphenol-A
PCBs: Polychlorinated biphenyl
NO_2_: Nitrogen dioxide
SO_2_: Sulfur dioxide
CO: Carbon monoxide
O_3_: Ozone
PM: Particulate matter
OCP: Oral contraceptive
ACR: American College of Radiology
BI-RADS: Breast Imaging-Reporting and Data System
AQCC: Air Quality Control Company
WHO: World Health Organization
EPA: Environmental Protection Agency
IDW: Inverse distance weighting
SD: Standard deviation
OR: Odds ratio
CI: Confidence interval
BMI: Body mass index
HR: Hazard ratio
EDCs: Endocrine-disrupting chemicals
PAHs: Polycyclic aromatic hydrocarbons

## Supplementary information

**Additional file 1: Supplementary Table 1-** Demographic, medical and drug history of study women with different breast densities. **Supplementary Table 2-** Comparison of pollutions criteria in women with high and low breast density. **Supplementary Table 3-** Comparison of pollutants criteria in women with different breast densities. **Supplementary table 4-** Evaluation the impact of pollutants on mammographic breast density with stepwise and non-stepwise logistic regression separately in menopause status. **Supplementary table 5-** Evaluation the impact of pollutants on mammographic breast density with stepwise and non-stepwise logistic regression separately in different age.

## Declaration

### Availability of data and material

The datasets used and analyzed during the current study are available on reasonable request from the corresponding author.
