# Supplementary material for: Air pollution exposure and mammographic breast density in Tehran, Iran: a cross-sectional study
Source: Environ Health Prev Med. 2022 Jul 1;27:28. doi: 10.1265/ehpm.22-00027 (PMC9283909; doi:10.1265/ehpm.22-00027)
Supplement: Supplementary file 1 — Additional file 1: Supplementary Table 1- Demographic, medical and drug history of study women with different breast densities. Supplementary Table 2- Comparison of pollutions criteria in women with high and low breast density. Supplementary Table 3- Comparison of pollutants criteria in women with different breast densities. Supplementary table 4- Evaluation the impact of pollutants on mammographic breast density with stepwise and non-stepwise logistic regression separately in menopause status. Supplementary table 5- Evaluation the impact of pollutants on mammographic breast density with stepwise and non-stepwise logistic regression separately in different age. [file ehpm-27-028-s001.docx]

**Supplementary Table 1- Demographic, medical and drug history of study women with different breast densities.**

| **Variables** | | **Grade A** | **Grade B** | **Grade C** | **Grade D** | **P-value** |
| --- | --- | --- | --- | --- | --- | --- |
| **Age** (years) | | 54.70 ± 8.14 | 52.49 ± 8.29 | 48.44 ± 6.48 | 46.10 ± 6.00 | <0.001 |
| **Body mass index** (Kg/m^2^) | | 31.60 ± 5.06 | 28.86 ± 5.27 | 27.39 ± 4.01 | 24.98 ± 5.14 | <0.001 |
| **Age of menarche** (years) | | 13.30 ±1.40 | 13.78 ± 1.63 | 13.49 ± 1.48 | 13.67 ± 1.62 | 0.359 |
| **Age at first birth** (years) | | 21.21± 5.48 | 21.39 ± 5.08 | 22.59 ± 6.58 | 25.55 ± 14.18 | 0.004 |
| **Parity** (n) | | 2.84 ± 1.60 | 2.45 ± 1.56 | 1.98 ± 1.31 | 1.62 ± 1.08 | <0.001 |
| **Breastfeeding duration** (month) | | 36.41 ± 36.95 | 34.47 ± 29.96 | 33.40 ± 29.41 | 25.63 ± 27.43 | 0.290 |
| **Menopause** | No | 23 (5.8) | 70 (17.7) | 269 (67.9) | 34 (8.6) | <0.001 |
|  | Yes | 80 (20.3) | 126 (31.9) | 183 (46.3) | 6 (1.5) |  |
| **History of OCP** | No | 57 (11.6) | 103 (21.0) | 306 (62.3) | 25 (5.1) | <0.001 |
|  | Yes | 46 (15.3) | 93 (31.0) | 146 (48.7) | 15 (5.0) |  |
| **Smoking** | Never | 86 (12.0) | 175 (24.4) | 417 (58.2) | 38 (5.3) | 0.016 |
|  | Active or passive | 17 (22.7) | 21 (28.0) | 35 (46.7) | 2 (2.7) |  |
| **Occupation** | Housewife | 97 (14.5) | 167 (24.9) | 374 (55.7) | 33 (4.9) | 0.008 |
|  | Practitioner | 3 (3.4) | 17 (19.5) | 60 (69.0) | 7 (8.0) |  |
|  | Retired | 3 (9.1) | 12 (36.4) | 18 (54.5) | 0 (0.0) |  |
| **Metformin** | No | 79 (11.3) | 171 (24.4) | 416 (59.4) | 34 (4.9) | 0.001 |
|  | Yes | 24 (26.4) | 25 (27.5) | 36 (39.6) | 6 (6.6) |  |
| **Aspirin** | No | 78 (11.4) | 161 (23.5) | 412 (60.1) | 34 (5.0) | <0.001 |
|  | Yes | 25 (23.6) | 35 (33.0) | 40 (37.7) | 6 (5.7) |  |
| **Calcium** | No | 53 (12.0) | 90 (20.5) | 271 (61.6) | 26 (5.9) | 0.001 |
|  | Yes | 50 (14.2) | 106 (30.2) | 181 (51.6) | 14 (4.0) |  |
| **Vitamin D** | No | 57 (14.3) | 107 (26.9) | 212 (53.3) | 22 (5.5) | 0.047 |
|  | Yes | 46 (11.7) | 89 (22.6) | 240 (61.1) | 18 (4.6) |  |
| **Vitamin E** | No | 90 (14) | 157 (24.3) | 368 (57.1) | 30 (4.7) | 0.547 |
|  | Yes | 13 (8.9) | 39 (26.7) | 84 (57.5) | 10 (6.8) |  |
| **Primrose** | No | 101 (13.4) | 186 (24.7) | 428 (56.9) | 37 (4.9) | 0.353 |
|  | Yes | 2 (5.1) | 10 (25.6) | 24 (61.1) | 3 (7.7) |  |
| **Omega-3** | No | 87 (12.5) | 177 (25.4) | 399 (57.3) | 33 (4.7) | 0.896 |
|  | Yes | 16 (17) | 19 (20.2) | 52 (55.3) | 7 (7.4) |  |
| **History of Breast Disease** | No | 81 (13.3) | 136 (22.3) | 361 (59.3) | 31 (5.1) | 0.021 |
|  | Yes | 22 (12.1) | 60 (33.0) | 91 (50.0) | 9 (4.9) |  |

Continuous variables present as mean ± standard deviation and categorical variables present as number with percentages in parenthesis. P-values were computes with t test for continues and chi square test for categorical variables.

**Supplementary Table 2- Comparison of pollutions criteria in women with high and low breast density.**

|  | **Low density (n=299)** | | | **High density (n=492)** | | | **P-value** |
| --- | --- | --- | --- | --- | --- | --- | --- |
|  | **Mean** | **SD** | **Median** | **Mean** | **SD** | **Median** |  |
| **CO**(ppm) | 1.00 | 0.77 | 0.90 | 0.90 | 0.60 | 0.90 | 0.054 |
| **O_3_**(ppb) | 6.73 | 4.60 | 6.80 | 6.47 | 4.07 | 6.80 | 0.404 |
| **NO_2_**(ppb) | 25.44 | 17.52 | 27.20 | 24.80 | 16.15 | 27.20 | 0.601 |
| **SO_2_**(ppb) | 4.29 | 3.24 | 4.00 | 3.97 | 2.64 | 4.00 | 0.125 |
| **PM_10_**(µg/m^3^) | 35.73 | 26.18 | 34.50 | 33.62 | 22.79 | 34.50 | 0.233 |
| **PM_2.5_**(µg/m^3^) | 17.25 | 12.60 | 17.00 | 16.35 | 11.04 | 17.00 | 0.295 |

P-values refer to t-test.

**Supplementary Table 3- Comparison of pollutants criteria in women with different breast densities.**

|  | **Low density (n=299)** | | **High density (n=492)** | | **P-value** |
| --- | --- | --- | --- | --- | --- |
|  | **Grade A** | **Grade B** | **Grade C** | **Grade D** |  |
|  | (N = 103) | (N = 196) | (N = 452) | (N = 40) |  |
| **CO**(ppm) | 0.96 ± 0.75 | 1.01 ± 0.79 | 0.91 ± 0.61 | 0.84 ± 0.58 | 0.216 |
| **O_3_**(ppb) | 6.25 ± 4.28 | 6.98 ± 4.76 | 6.51 ± 4.09 | 5.97 ± 3.88 | 0.359 |
| **NO_2_**(ppb) | 23.9 ± 16.59 | 26.25 ± 17.98 | 25.03 ± 16.24 | 22.19 ± 15.05 | 0.443 |
| **SO_2_**(ppb) | 3.99 ± 2.97 | 4.45 ± 3.37 | 3.98 ± 2.68 | 3.8 ± 2.27 | 0.239 |
| **PM_10_**(µg/m^3^) | 33.28 ± 24.37 | 37.02 ± 27.06 | 33.81 ± 23.02 | 31.43 ± 20.14 | 0.334 |
| **PM_2.5_**(µg/m^3^) | 16.06 ± 11.57 | 17.87 ± 13.09 | 16.45 ± 11.14 | 15.27 ± 9.87 | 0.378 |

Data was presented as Mean ± SD and compared with ANOVA.

Supplementary table 4- Evaluation the impact of pollutants on mammographic breast density with stepwise and non-stepwise logistic regression separately in menopause status.

|  | **Non-stepwise algorithm** | | | | **Stepwise algorithm** | | | |
| --- | --- | --- | --- | --- | --- | --- | --- | --- |
|  | **P-value** | **OR** | **95%C.I Lower** | **95%C.I Upper** | **P-value** | **OR** | **95%C.I Lower** | **95%C.I Upper** |
| **Menopause**  **No (n = 396)** | |  |  |  |  |  |  |  |
| CO (ppm) | 0.733 | 0.791 | 0.205 | 3.047 | 0.144 | 0.450 | 0.154 | 1.314 |
| NO_2_ (ppb) | 0.135 | 1.258 | 0.931 | 1.701 | 0.105 | 1.035 | 0.993 | 1.079 |
| O_3_ (ppb) | 0.859 | 0.993 | 0.913 | 1.079 |  |  |  |  |
| SO_2_ (ppb) | 0.124 | 0.683 | 0.419 | 1.111 |  |  |  |  |
| PM_10_ (µg/m^3^) | 0.498 | 0.920 | 0.723 | 1.171 |  |  |  |  |
| PM_2.5_ (µg/m^3^) | 0.337 | 1.234 | 0.803 | 1.897 |  |  |  |  |
| Constant | <0.001 | 2.765 |  |  | <0.001 | 2.892 |  |  |
| **Yes (n = 395)** | |  |  |  |  |  |  |  |
| CO (ppm) | **0.041** | 0.338 | 0.120 | 0.955 | **0.013** | 0.314 | 0.125 | 0.785 |
| NO_2_ (ppb) | 0.214 | 0.885 | 0.729 | 1.073 | **0.039** | 1.039 | 1.002 | 1.077 |
| O_3_ (ppb) | **0.041** | 1.069 | 1.003 | 1.139 |  |  |  |  |
| SO_2_ (ppb) | 0.910 | 0.977 | 0.653 | 1.462 |  |  |  |  |
| PM_10_ (µg/m^3^) | 0.972 | 1.003 | 0.844 | 1.193 |  |  |  |  |
| PM_2.5_ (µg/m^3^) | 0.988 | 0.998 | 0.727 | 1.369 |  |  |  |  |
| Constant | 0.585 | 1.106 |  |  | 0.743 | 1.060 |  |  |

OR = Odds ratio, C.I = Confidence interval, NO_2_= Nitrogen dioxide (NO_2_), SO_2_ = Sulfur dioxide, CO = Carbon monoxide, O_3_ = Ozone, PM = Particulate matter

Supplementary table 5- Evaluation the impact of pollutants on mammographic breast density with stepwise and non-stepwise logistic regression separately in different age.

|  | **Non-stepwise algorithm** | | | | **Stepwise algorithm** | | | |
| --- | --- | --- | --- | --- | --- | --- | --- | --- |
|  | **P-value** | **OR** | **95%C.I Lower** | **95%C.I Upper** | **P-value** | **OR** | **95%C.I Lower** | **95%C.I Upper** |
| **Age**  **≤45 (n = 240)** |  |  |  |  |  |  |  |  |
| CO (ppm) | 0.119 | 0.208 | 0.029 | 1.500 | **0.033** | 0.148 | 0.026 | 0.856 |
| NO_2_ (ppb) | 0.505 | 1.119 | 0.803 | 1.560 | **0.020** | 1.084 | 1.013 | 1.160 |
| O_3_ (ppb) | 0.140 | 1.084 | 0.974 | 1.207 |  |  |  |  |
| SO_2_ (ppb) | 0.141 | 0.643 | 0.358 | 1.157 |  |  |  |  |
| PM_10_ (µg/m^3^) | 0.416 | 1.172 | 0.799 | 1.720 |  |  |  |  |
| PM_2.5_ (µg/m^3^) | 0.455 | 0.758 | 0.366 | 1.569 |  |  |  |  |
| Constant | 0.001 | 2.941 |  |  | <0.001 | 3.116 |  |  |
| **>45 (n = 551)** |  |  |  |  |  |  |  |  |
| CO (ppm) | 0.123 | 0.534 | 0.241 | 1.185 | **0.016** | 0.418 | 0.206 | 0.848 |
| NO_2_ (ppb) | 0.737 | 0.969 | 0.807 | 1.163 | **0.041** | 1.029 | 1.001 | 1.059 |
| O_3_ (ppb) | 0.191 | 1.038 | 0.982 | 1.097 |  |  |  |  |
| SO_2_ (ppb) | 0.766 | 0.947 | 0.661 | 1.357 |  |  |  |  |
| PM_10_ (µg/m^3^) | 0.351 | 0.931 | 0.800 | 1.082 |  |  |  |  |
| PM_2.5_ (µg/m^3^) | 0.285 | 1.160 | 0.884 | 1.523 |  |  |  |  |
| Constant | 0.076 | 1.322 |  |  | 0.067 | 1.325 |  |  |

OR = Odds ratio, C.I = Confidence interval, NO_2_= Nitrogen dioxide (NO_2_), SO_2_ = Sulfur dioxide, CO = Carbon monoxide, O_3_ = Ozone, PM = Particulate matter
